# Neoadjuvant chemotherapy with trastuzumab in HER2-positive breast cancer: pathologic complete response rate, predictive and prognostic factors

**DOI:** 10.1590/1414-431X20165674

**Published:** 2017-01-26

**Authors:** I.P.C. Buzatto, A. Ribeiro-Silva, J.M. Andrade, H.H.A. Carrara, W.A. Silveira, D.G. Tiezzi

**Affiliations:** 1 Setor de Mastologia, Departamento de Ginecologia e Obstetrícia, Faculdade de Medicina de Ribeirão Preto, Universidade de São Paulo, Ribeirão Preto, SP, Brasil; 2 Departamento de Patologia, Faculdade de Medicina de Ribeirão Preto, Universidade de São Paulo, Ribeirão Preto, SP, Brasil

**Keywords:** Neoadjuvant chemotherapy, Trastuzumab, Pathologic complete response, Predictive factors of response, Prognosis

## Abstract

The purpose of this study was to retrospectively review the pathologic complete response (pCR) rate from patients (n=86) with stage II and III HER2-positive breast cancer treated with neoadjuvant chemotherapy at our institution from 2008 to 2013 and to determine possible predictive and prognostic factors. Immunohistochemistry for hormone receptors and Ki-67 was carried out. Clinical and pathological features were analyzed as predictive factors of response to therapy. For survival analysis, we used Kaplan-Meier curves to estimate 5-year survival rates and the log-rank test to compare the curves. The addition of trastuzumab to neoadjuvant chemotherapy significantly improved pCR rate from 4.8 to 46.8%, regardless of the number of preoperative trastuzumab cycles (P=0.0012). Stage II patients achieved a higher response rate compared to stage III (P=0.03). The disease-free and overall survivals were not significantly different between the group of patients that received trastuzumab in the neoadjuvant setting (56.3 and 70% at 5 years, respectively) and the group that initiated it post-operatively (75.8 and 88.7% at 5 years, respectively). Axillary pCR post neoadjuvant chemotherapy with trastuzumab was associated with reduced risk of recurrence (HR=0.34; P=0.03) and death (HR=0.21; P=0.02). In conclusion, we confirmed that trastuzumab improves pCR rates and verified that this improvement occurs even with less than four cycles of the drug. Hormone receptors and Ki-67 expressions were not predictive of response in this subset of patients. Axillary pCR clearly denotes prognosis after neoadjuvant target therapy and should be considered to be a marker of resistance, providing an opportunity to investigate new strategies for HER2-positive treatment.

## Introduction

Breast cancer is the most common malignancy among women all over the world, and it is considered a heterogeneous disease. Around 25% of all breast cancer overexpress the human epidermal growth factor receptor 2 (HER2), and is traditionally associated with worse prognosis (1). The activation of HER2 occurs through dimerization with other proteins of the family, triggering multiple downstream pathways required for the abnormal proliferation of cancer cells (2).

Neoadjuvant chemotherapy is the standard of care for patients with locally advanced breast cancer. Even though it does not enhance overall survival when compared with adjuvant therapy, neoadjuvant chemotherapy has some secondary benefits (1). It can contribute to surgical downstaging and increase rates of breast-conserving surgery, in addition to providing an *in vivo* assessment of tumor response to chemotherapy (3).

Trastuzumab is a humanized monoclonal antibody that binds to the extracellular domain of HER2. It can potently suppress cancer cells’ growth, proliferation, and survival in direct and indirect manners (2). It is well known that trastuzumab has survival benefits when associated with chemotherapy in the treatment of patients with early operable and metastatic HER2-positive breast cancer (4–6). In the neoadjuvant setting, when associated with chemotherapy, trastuzumab significantly improved pathologic complete response, and reduced the risk of relapse, disease progression and death when compared to chemotherapy alone (4). However, the available studies compared patients that received neoadjuvant chemotherapy with trastuzumab to patients that did not receive trastuzumab at all or with HER2-negative tumors (4,7). It is necessary to investigate if initiating trastuzumab in the neoadjuvant setting has the same survival benefits when compared to patients that used it postoperatively.

Pathologic complete response (pCR) is usually defined as absence of residual invasive cancer in breast and lymph nodes in surgical specimens after neoadjuvant therapy. It is considered an important prognostic factor and is associated with long-term survival. pCR has been adopted as the primary end point for neoadjuvant trials and reaches up to 78% in patients treated with trastuzumab (3,7). However, a significant fraction of these patients eventually relapse or develop progressive disease. This suggests that these tumors have intrinsic or acquired mechanisms of resistance to targeted therapies (8).

In this article, we reviewed the rate of pCR from patients with HER2-positive breast cancer treated with neoadjuvant chemotherapy at our institution and investigated possible clinical, histological and immunohistochemical predictive and prognostic factors. This knowledge would allow a better selection of patients that could benefit from therapy with trastuzumab. In addition, costs and adverse events could be minimized, leading to an individualized cancer treatment (9).

## Patients and Methods

This cohort study with retrospective data collection evaluated clinical, histological, and immunohistochemical characteristics of all patients with histologically confirmed HER2-positive, stage II and III breast cancer, treated with neoadjuvant chemotherapy between 2008 and 2013 at our institution. The Institutional Review Board of the Hospital das Clínicas, Faculdade de Medicina de Ribeirão Preto, SP, Brazil, approved this study (#497.095).

### Procedures

The HER2 status was assessed by immunohistochemistry (IHC) and tumors were considered HER2-positive when IHC staining was 3+ (uniform, intense membrane staining of >30% of invasive tumor cells) and 2+ (uniform, weak membrane staining of >30% of invasive tumor cells), in which the HER2 gene amplification was confirmed by chromogenic *in situ* hybridization (CISH).

We used microscopically selected representative samples from breast tissues stored in our Pathology department. A paraffin tissue microarray (TMA) block was built to minimize experimental variability and reduce costs. We used the Tissue Microarray Builder Kit (Histopathology Ltd., Hungary) to arrange 24 cylinders of 2 mm^2^ in one recipient paraffin block.

Immunohistochemical staining for estrogen receptor, progesterone receptor and Ki-67 was performed in the TMA sections using mouse monoclonal antibody at 1:200, 1:200 and 1:100 dilutions, respectively (NovoCastra, UK*)*.

To quantify the expression of Ki-67 we scanned the slides with the automated scanning system Aperio XT (Aperio Technologies, USA), and used the software ImageScope (USA) for cell count. A 14% cut-off was used to define a high expression of Ki-67. At least 500 contiguous tumor cells were counted to establish the index.

In order to confirm the amplification of the HER2 gene we performed a dual color chromogenic (CISH) assay using the Kit CISH™ HER2 SPOT-Light¯, Zymed (Invitrogen, USA) in the TMA sections. The interpretation followed the College of American Pathologists guidelines. The amplification was confirmed in all 41 tumors tested.

### Treatment

Patients were treated with neoadjuvant chemotherapy with anthracyclines and/or taxanes, for at least 4 cycles, according to the current protocol. The 86 patients included in the study were divided into two groups: 65 patients received neoadjuvant chemotherapy with trastuzumab followed by adjuvant trastuzumab while 21 patients received neoadjuvant chemotherapy alone followed by adjuvant trastuzumab.

The chemotherapy regimens used were: 1) 4 cycles of 75 mg/m^2^ epirubicin + 500–600 mg/m^2^ cyclophosphamide followed by 4 cycles of 100 mg/m^2^ docetaxel every 21 days +/- trastuzumab; 2) 4 to 6 cycles of 80–100 mg/m^2^ docetaxel + trastuzumab every 21 days; 3) 6 cycles of 500 mg/m^2^ fluorouracil + 75 mg/m^2^ epirubicin + 500 mg/m^2^ cyclophosphamide every 21 days; 4) 4 cycles of 75 mg/m^2^ docetaxel + 60 mg/m^2^ epirubicin every 21 days. All patients completed 1 year of treatment with adjuvant trastuzumab, except those that developed severe toxicity.

### Statistical analyses

Disease-free survival was considered as the length of time between the surgery and the first recurrence event (local or distant). Overall survival was considered as the length of time between the cancer diagnosis and death. Patients that were lost to follow-up or did not reach any event (recurrence or death) were censored in the analysis.

Statistical analyses were done with GraphPad Prism 6 software (USA). To analyze the categorical variables, we used Pearson chi-square test or Fisher's exact test. For survival analysis, we used Kaplan-Meier curves to estimate 5-year survival rates and the log-rank test to compare the curves. Statistical significance was assumed at a 0.05 level (P<0.05).

## Results

All of the 86 patients were treated with adjuvant trastuzumab, 66 completed 1 year of adjuvant treatment, 8 developed cardiac toxicity severe enough to interrupt the drug (9.5%), while 11 were still under treatment with trastuzumab at the time of the study. One patient from the first group died during the neoadjuvant treatment from febrile neutropenia and, therefore, did not receive surgical or adjuvant treatment.

The addition of trastuzumab to the neoadjuvant chemotherapy significantly improved the pCR rate. Of the 64 patients that received at least one cycle of trastuzumab in the neoadjuvant setting, 30 achieved pCR (46.8%), while in the group of 21 patients treated with neoadjuvant chemotherapy without trastuzumab only 1 achieved pCR (4.8%).

In a subgroup analysis, we evaluated if the number of trastuzumab cycles received preoperatively influenced the pCR rate. Of the 65 patients treated with neoadjuvant chemotherapy with trastuzumab, 46 received at least 4 cycles of trastuzumab (T group) and 19 received only 1 to 3 cycles of the drug together with neoadjuvant chemotherapy (Chemo/T group). As can be seen in Table 1, the improvement in the pCR rate was consistent regardless of the number of trastuzumab cycles received preoperatively. The baseline characteristics of the patients are also shown in Table 1. The groups were similar in age, clinical tumor stage at diagnosis, tumor grade and positivity to hormone receptors.

**Table:**
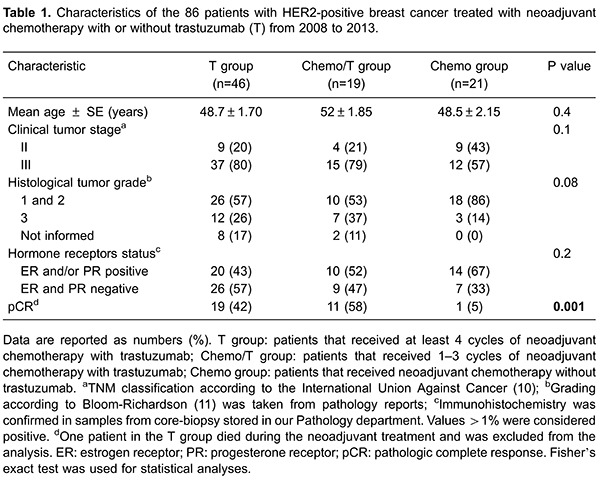

The rates of breast-conserving surgery were not significantly influenced by the addition of trastuzumab nor was pCR. Of patients treated with neoadjuvant chemotherapy with trastuzumab, 33% had conservative surgery, compared with 52% from the chemotherapy alone (P*=*0.1). In the group of patients that achieved pCR, 35% underwent breast-conserving surgery, while in the group with residual tumor in the breast this rate was 39% (P*=*0.8).

**Figure 1:**
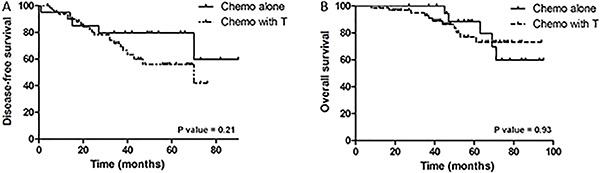
. Disease-free (*A*) and overall (*B*) survival of the 86 patients with HER2-positive breast cancer treated with neoadjuvant chemotherapy (Chemo) with or without trastuzumab (T). All patients received adjuvant treatment with trastuzumab. Log-rank test was used to compare the curves.

The disease-free and overall survival (Figure 1) were not significantly different between the group of patients that started trastuzumab in the neoadjuvant setting and the group treated with neoadjuvant chemotherapy alone and adjuvant trastuzumab (56.3 *vs* 75.8% at 5 years, P=0.38, and 70 *vs* 88.7% at 5 years, P=0.26, respectively).

We investigated clinical, histological and immunohistochemical predictive factors for response to neoadjuvant chemotherapy with trastuzumab in the 65 patients treated with at least one cycle of the drug preoperatively (Table 2). The only predictive factor for pCR in our sample was the clinical stage at diagnosis; 75% of the patients in stage II achieved pCR, compared to 40% in stage III (P=0.03). Negativity to hormone receptor, histological high grade and high Ki-67 were not predictive factors for pCR.

**Table:**
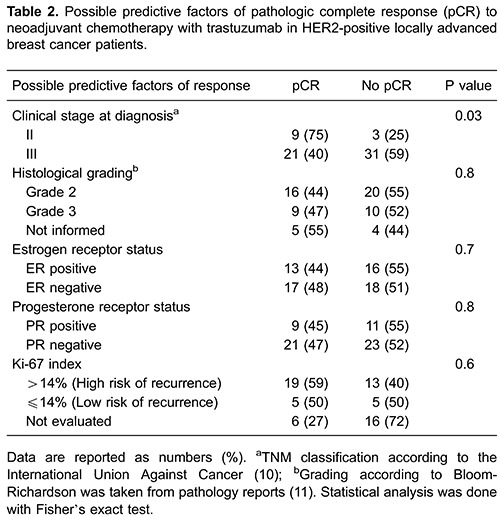

**Figure 2:**
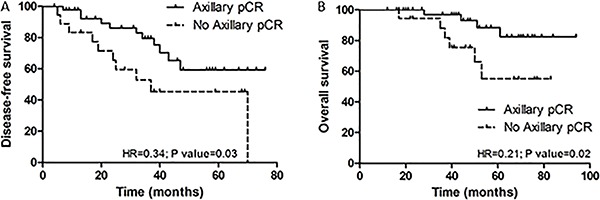
Disease-free (*A*) and overall (*B*) survival of the 63 patients with HER2-positive breast cancer treated with neoadjuvant chemotherapy with trastuzumab by axillary pathologic complete response (pCR) status. One patient died during neoadjuvant chemotherapy and was excluded from the analyses and 1 patient was excluded because the data of axillary involvement was not available. Log-rank test was used to compare the curves. HR: hazard ratio.

We evaluated possible prognostic factors in our population of HER2-positive breast cancer patients treated with neoadjuvant chemotherapy associated with trastuzumab. pCR is considered an important prognostic factor and is associated with better disease-free and overall survival (12). pCR (no invasive residual tumor in breast and axilla) was not a statistically significant prognostic factor in our study. However, pCR in the axilla (no residual tumor in the lymph nodes, regardless to the response in the breast) was an independent prognostic factor (Figure 2). The 5-year disease-free survival was 59.4% in the group of patients with axillary pCR and 45.7% in the group with residual burden in the axilla (P=0.019). The overall survival in the group of patients with axillary pCR was 88% while in the group without axillary pCR was 55% (P=0.04).

## Discussion

Our findings confirmed that the addition of trastuzumab to neoadjuvant chemotherapy significantly improves pCR rates. This is consistent with other trials that demonstrated that 1 year of trastuzumab starting as neoadjuvant almost doubled pCR rates (4). The ideal number of trastuzumab cycles to be done preoperatively is not well established in the literature. A German study published in 2011 did not observe better pCR rates in patients receiving more than 4 cycles of neoadjuvant trastuzumab (13). Our study suggests that the improvement in pCR rates persists even with less than four cycles of the drug.

In a meta-analysis published in 2011, even though the probability to achieve pCR was higher in the trastuzumab plus chemotherapy group (HR 1.85; P<0.001), breast-conserving surgery rates were similar to the group that used chemotherapy alone (3). Our study had similar findings; the rates of breast conserving surgery were not influenced by the addition of trastuzumab or by pCR rates. The low rate of conservative surgery in the present study (38%) may be a reflex of the high proportion of tumors with skin involvement (34% of T4 tumors). The subgroup analysis with only stage II tumors found 66% of conservative surgeries.

The 3 major trials that evaluated neoadjuvant treatment with trastuzumab had a limitation in the survival analyses, because they compared it with patients that did not receive adjuvant trastuzumab and patients with HER2 negative tumors (4,14,15). Our study demonstrated that neoadjuvant trastuzumab is equivalent to adjuvant trastuzumab. This result is in agreement with several trials that compared neoadjuvant to adjuvant chemotherapy; in general, the moment of administration of systemic therapy in breast cancer does not affect survival (16,17).

Even though trastuzumab presented a great improvement in the treatment of HER2-positive breast cancer, it is associated with relevant adverse cardiac events and with significantly elevated cost of treatment (9,18,19). In our study, 9.5% of patients had to interrupt the drug because of cardiac toxicity. This has increased the interest in identifying predictive factors of response (20). We investigated clinical, histological and immunohistochemical predictive factors of response to neoadjuvant chemotherapy with trastuzumab and found statistical significance only for clinical stage at diagnosis.

Our findings reinforce the importance of early diagnoses in some aspects. Only 22 of the 86 patients were at stage II disease, but they had significantly greater chance of achieving pCR and having conservative surgery when compared to the patients at stage III. The relevance of the TNM staging has been questioned in the last few years (21). A recent study suggests that the biomarkers add important predictive and prognostic information to the TNM staging, especially in locally advanced tumors, but they do not replace it (21). Our data confirms this finding. Among the routinely used biomarkers (hormone receptor, Ki-67, histological grade), none was capable to predict pCR, except for the clinical stage at diagnosis.

We evaluated possible prognostic factors in our population of HER2-positive breast cancer patients treated with neoadjuvant chemotherapy associated with trastuzumab. Several studies have demonstrated a relation between the number of positive nodes in the axilla after neoadjuvant treatment and survival (22–25). Our data suggest that axillary pCR might be the most important independent prognostic factor and residual disease in the axilla should be considered a marker of resistance, providing an opportunity to investigate new strategies for HER2-positive treatment.
